# Lipids determine the toxicity of human islet polypeptide aggregates *in vivo*

**DOI:** 10.1016/j.jbc.2024.108029

**Published:** 2024-11-29

**Authors:** Jadon Sitton, Davis Pickett, Axell Rodriguez, Dmitry Kurouski

**Affiliations:** 1 Department of Biochemistry and Biophysics, Texas A&M University, College Station, Texas, United States; 2 Department of Biomedical Engineering, Texas A&M University, College Station, Texas, United States

**Keywords:** hIAPP, phospholipids, oligomers, fibrils, toxicity, *C. elegans*

## Abstract

The onset and progression of type 2 diabetes is linked to the accumulation and aggregation of human islet amyloid polypeptide (hIAPP) in the pancreas. Amyloid oligomers and fibrils formed as a result of such aggregation exert high cytotoxicity. Although some pieces of evidence suggest that lipids could alter the rate of hIAPP aggregation, the effect of lipids on the aggregation properties of this peptide remains unclear. In this study, we investigate the effect of sphingophospholipid and anionic and zwitterionic phospholipids with different lengths of fatty acids on the aggregation of hIAPP. We found that anionic lipids drastically accelerate peptide aggregation, whereas this effect was substantially weaker for sphingophospholipid and zwitterionic phospholipid. Biophysical analysis revealed that the presence of lipids resulted in substantial differences in morphology and secondary structure of hIAPP fibrils compared to the protein aggregates grown in the lipid-free environment. We also found that zwitterionic phospholipids drastically increased cytotoxicity of hIAPP aggregates, whereas this effect was less evident for sphingophospholipid and anionic phospholipid. Our results showed that drastic differences in lipid-determined cytotoxicity of hIAPP aggregates were linked to molecular mechanisms of autophagy, exocytosis, and unfolded protein response. These findings suggest that molecular candidates that could disrupt protein–lipid interactions would allow for deceleration of the onset and progression of type 2 diabetes.

Type 2 diabetes affects over 520 million people worldwide (1). This chronic pathology is characterized by irreversible insulin resistance which ultimately leads to chronic hyperglycemia (2). One of the hallmarks of type 2 diabetes is the progressive accumulation and aggregation of human islet amyloid polypeptide (hIAPP), a small 37 amino acid peptide hormone secreted by the pancreas (3). Together with insulin, IAPP maintains glucose homeostasis, controls gastric emptying, and suppresses glucagon secretion (4). Under hyperglycemic conditions, high concentrations of hIAPP facilitate its aggregation into amyloid oligomers and fibrils. These protein aggregates exert high cytotoxicity causing progressive death of insulin-secreting pancreatic islet beta cells, which in turn further accelerates the progression of diabetes (5). hIAPP oligomers and fibrils could also trigger aggregation of other proteins in the brain, such as amyloid β (Aβ) and α-synuclein (α-syn). These findings point to a connection between hIAPP aggregation and neurodegeneration (6, 7, 8).

A growing number of studies indicate that lipid membranes can drastically alter the aggregation properties of amyloidogenic proteins. For instance, Chi *et al.* found that amyloid-β 1 to 42 (Aβ_1-42_) interacts with anionic and zwitterionic membranes composed; however, this interaction is amplified in anionic lipids (9). Zhaliazka *et al.* demonstrated that cholesterol, phosphatidylcholine, and cardiolipin drastically accelerated the aggregation rate of Aβ and increased toxicity of oligomers and fibrils compared to Aβ aggregates formed in the lipid-free environment. Galvagnion *et al.* showed that lipids drastically altered rates of α-syn aggregation (10, 11, 12). Moreover, recent work has shown that lipids can alter the functionality of proteins like α-syn in their native form (13). Furthermore, our group found that the length and saturation of fatty acids (FAs) in phospholipids could drastically alter the aggregation properties of insulin, α-syn, and transthyretin (14, 15, 16, 17). Additionally, membrane hydrophobic thickness has also been observed to have an astute effect on amyloid formation, with reduced bilayer thickness having been observed to stabilize globular oligomers of Aβ in contrast to thicker membranes (18). Free lipids in solution below their critical micelle concentration have been observed to catalyze the initial stages of aggregation through dynamics with the lipid membrane (19). Together, previous studies have shown that characteristics of both lipids and lipid membranes significantly impact the formation and toxicity of amyloid proteins.

Similar observations were reported by Zhang *et al.* for hIAPP (20). It was found that at low concentrations, anionic lipids facilitated hIAPP aggregation. At the same time, cholesterol at or below physiological levels significantly decelerated hIAPP aggregation, as well as lowered the propensity of hIAPP aggregates to cause membrane leakages. Nanga *et al*. also resolved a predominantly helical conformation of hIAPP in the presence of SDS micelles with NMR spectroscopy, in contrast to the intrinsically disordered structure of monomeric native hIAPP, effectively showing hIAPP undergoes specific conformational changes in the presence of negatively charged membranes (21). Moreover, Brender *et al*. found that in the absence of the amyloidogenic core, hIAPP conformations are still able to disrupt membranes (22). This was further corroborated by Green *et al.* who observed that both human IAPP and rat IAPP can cause membrane lesions despite rat IAPP’s inability to form amyloid fibrils (23).This suggests that membrane-catalyzed conformational changes in hIAPP structure can disrupt cell membranes even in the absence of amyloid formation, further stressing the importance in understanding the influence of lipids on the cellular toxicity of IAPP. Lipid raft components have also been observed to interact with amyloid proteins, suggesting localized lipid domains within a membrane influence aggregation, and that individual lipids can have specific effects (24). This is further corroborated by Sciacca *et al.* who found cholesterol can suppress coil-to-helix transition in hIAPP and enhance the fibril-dependent membrane disruption of hIAPP aggregates (25). Soong *et al.* also observed a temperature-dependent membrane-catalyzed folding of hIAPP that preceded fibril formation, suggesting lipid membranes may also alter the misfolding cascade of hIAPP and by the same effect the toxicity (26). This evidence together supports the need for further analysis of hIAPP–lipid interactions, especially in terms of the cellular and organismal impacts of hIAPP aggregates when formed in different lipid environments.

In this study, we investigate the influence of six different saturated and unsaturated phospholipids and sphingolipids on the aggregation properties of hIAPP. Using a combination of biophysical methods, we determine the influence of lipid membranes on the morphology and secondary structure of IAPP fibrils. We also reveal the extent to which different lipids altered the toxicity of IAPP aggregates to both pancreatic β cell and neural cell lines, as well as altered the lifespan of *Caenorhabditis elegans*.

## Results

### Lipids accelerated the aggregation of hIAPP

**Figure 1:**
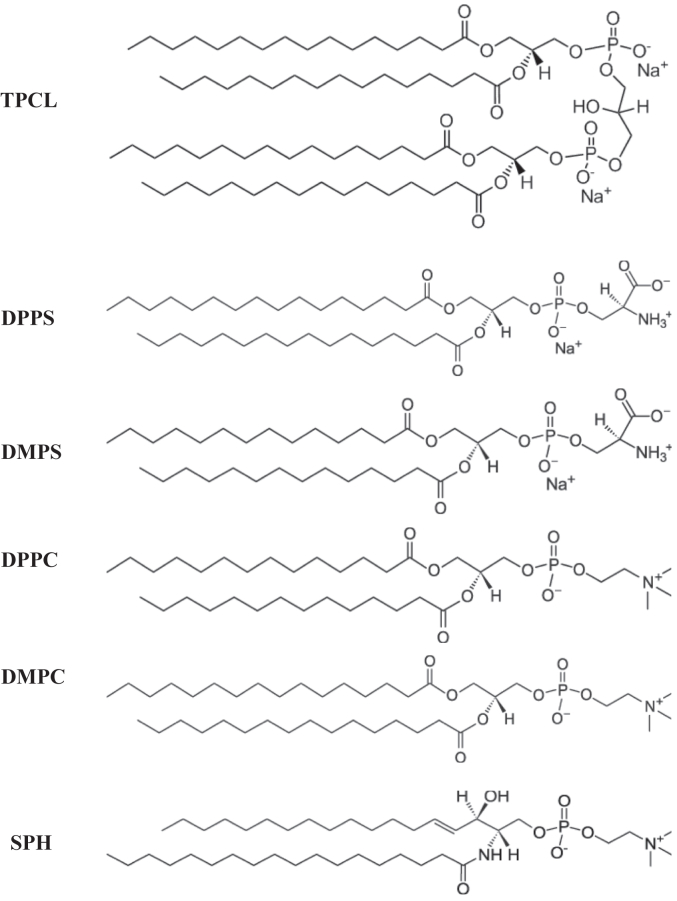
**Molecular structure of lipids of TPCL, DPPS, DMPS, DPPC, DMPC, SPH.** DPPS, 1,2-dipalmitoyl-sn-glycero-3-phospho-L-serine; DMPS, 1,2-dimyristoyl-sn-glycero-3-phospho-L-serine; DPPC, 1,2-dipalmitoyl-sn-glycero-3-phosphocholine; DMPC, 1,2-dimyristoyl-sn-glycero-3-phosphocholine; SPH, N-octadecanoyl-D-erythro-sphingosylphosphorylcholine; TPCL, 1′,3′-bis[1,2-dipalmitoyl-sn-glycero-3-phospho]-glycerol.

Utilizing thioflavin T (ThT) kinetic assays, we determined the aggregation properties of hIAPP in the presence and absence of an equimolar concentration of large unilamellar vesicles (LUVs) composed of 1′,3′-bis[1,2-dipalmitoyl-sn-glycero-3-phospho]-glycerol (TPCL), 1,2-dipalmitoyl-sn-glycero-3-phospho-L-serine (DPPS), 1,2-dimyristoyl-sn-glycero-3-phospho-L-serine (DMPS), 1,2-dipalmitoyl-sn-glycero-3-phosphocholine (DPPC), 1,2-dimyristoyl-sn-glycero-3-phosphocholine (DMPC), and N-octadecanoyl-D-erythro-sphingosylphosphorylcholine (SPH) (Fig. 1). Membrane packing and curvature has also been observed to affect amyloid aggregation, altering the unfolding and amyloid formation cascade (27). Small unilamellar vesicles were observed to have more curvature and less consistent packing compared to LUVs. Thus, we utilized LUVs to ensure the membrane curvature, and packing was consistent across samples.

**Figure 2:**
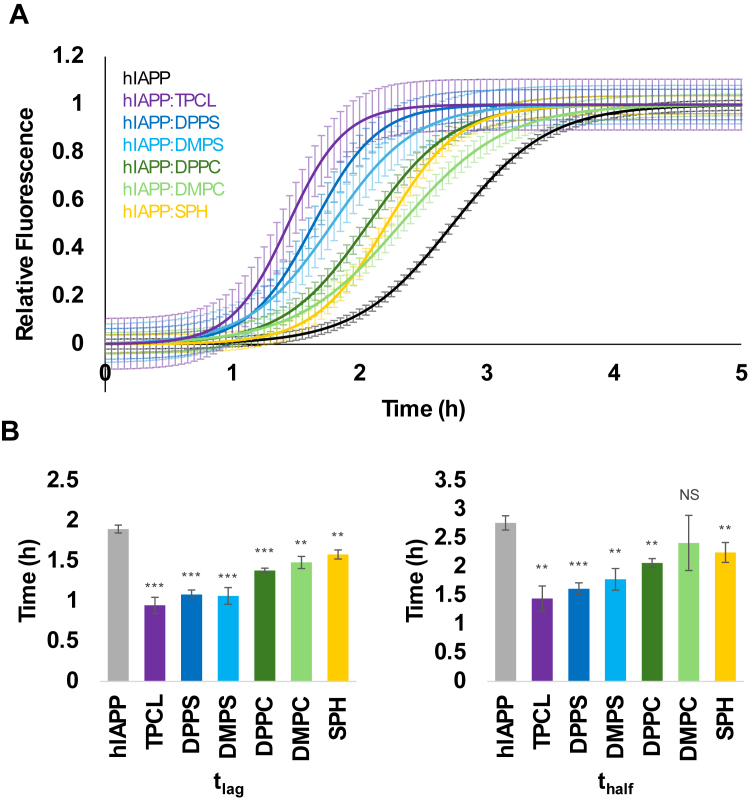
**Lipids alter the aggregation kinetics of hIAPP.***A* and *B*, ThT kinetics of hIAPP aggregation in the presence and absence of LUVs composed of different lipids with the corresponding t_lag_ and t_half_. Error bars represent confidence intervals for kinetics curves. Experiments were completed in triplicates. Significance calculated using Student’s *t* test. ∗∗*p* < 0.01, ∗∗∗*p* < 0.001. DMPC, 1,2-dimyristoyl-sn-glycero-3-phosphocholine; DMPS, 1,2-dimyristoyl-sn-glycero-3-phospho-L-serine; DPPC, 1,2-dipalmitoyl-sn-glycero-3-phosphocholine; DPPS, 1,2-dipalmitoyl-sn-glycero-3-phospho-L-serine; LUV, large unilamellar vesicle; hIAPP, human islet amyloid polypeptide; NS, not significant; SPH, N-octadecanoyl-D-erythro-sphingosylphosphorylcholine; ThT, thioflavin; TPCL, 1′,3′-bis[1,2-dipalmitoyl-sn-glycero-3-phospho]-glycerol.

In the lipid-free environment, hIAPP aggregated with a well-defined lag-phase (t_lag_) of 1.9 ± 0.05 h, whereas the presence of TPCL shortened t_lag_ to 0.95 ± 0.10 h, Fig. 2*B*. We also observed a strong decrease in t_lag_ for DPPS and DMPS (t_lag_ = 1.08 ± 0.06 and t_lag_ = 1.07 ± 0.10 h, respectively). At the same time, a much less substantial shortening of t_lag_ of hIAPP was observed for zwitterionic lipids, DPPC and DMPC (t_lag_ = 1.38 ± 0.03 h and t_lag_ = 1.48 ± 0.08 h, respectively). Finally, SPH exerted the weakest effect on t_lag_ of hIAPP aggregation (t_lag_ = 1.58 ± 0.06 h). These results indicate that as net negative charge of lipids increases, a decrease in t_lag_ of hIAPP aggregation is observed. Thus, the net charge of lipids is greatest determining factor in the rate of hIAPP oligomerization. It should be noted that we observed remarkably similar t_lag_ in the presence of DMPC and DPPC, as well as in the presence of DMPS and DPPS. These findings indicate that the length of FA tails has very little effect on the rate of hIAPP oligomerization.

The same conclusions could be made for the effect of lipids on the rate of hIAPP fibril formation and elongation. We found that anionic lipids [TPCL (t_half_ = 1.45 ± 0.22 h), DMPS (t_half_ = 1.78 ± 0.19 h), and DPPS (t_half_ = 1.62 ± 0.1 h)] strongly accelerated the rate of fibril formation. This effect was less pronounced for zwitterionic lipids [DMPC (t_half_ = 2.42 ± 0.48 h), DPPC (t_half_ = 2.07 ± 0.08 h), and SPH (t_half_ = 2.25 ± 0.17 h)]; however, an accelerating effect was still observed compared to hIAPP in a lipid-free environment (2.77 ± 0.13 h), Fig. 2*B*.

### The presence of lipid membranes changed the morphology and secondary structure of hIAPP fibrils

**Figure 3:**
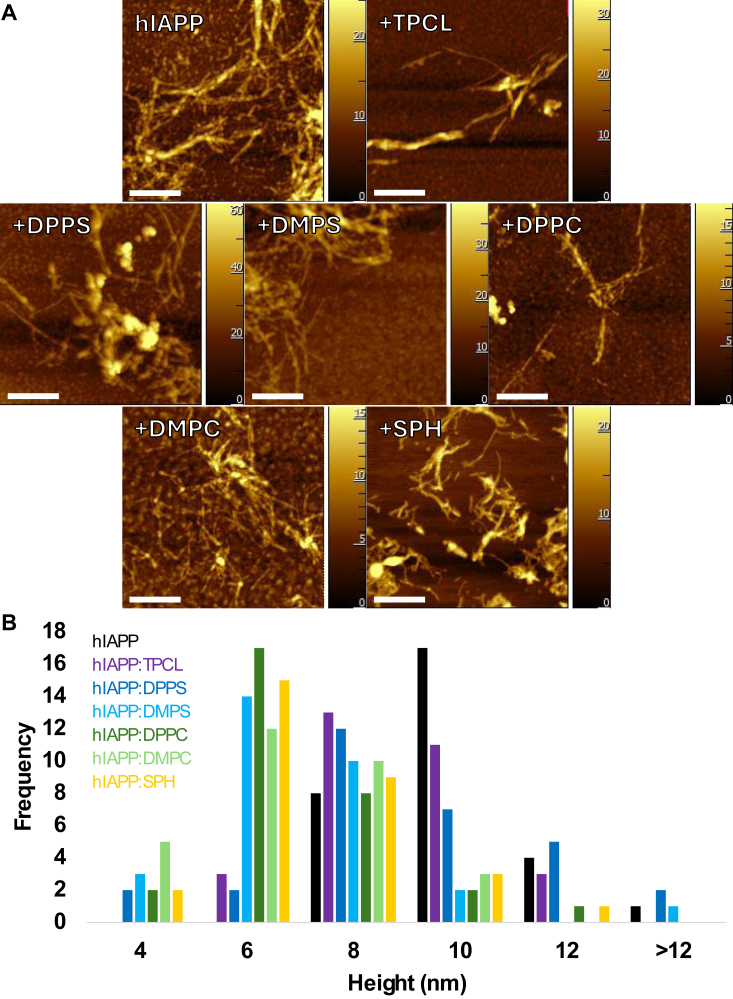
**Lipid membranes alter morphology hIAPP aggregates.***A*, AFM images of hIAPP aggregates formed in the presence and absence of LUVs composed of different lipids. *B*, histograms of hIAPP fibril heights based on n = 30 measurements. Scale bar = 500 nm. AFM, atomic force microscopy; DMPC, 1,2-dimyristoyl-sn-glycero-3-phosphocholine; DMPS, 1,2-dimyristoyl-sn-glycero-3-phospho-L-serine; DPPC, 1,2-dipalmitoyl-sn-glycero-3-phosphocholine; DPPS, 1,2-dipalmitoyl-sn-glycero-3-phospho-L-serine; hIAPP, human islet amyloid polypeptide; LUV, large unilamellar vesicle; SPH, N-octadecanoyl-D-erythro-sphingosylphosphorylcholine; TPCL, 1′,3′-bis[1,2-dipalmitoyl-sn-glycero-3-phospho]-glycerol.

Using atomic force microscopy (AFM), we investigated the extent to which net charge and length of FAs in lipids alter the morphology of hIAPP fibrils. We found that hIAPP fibrils formed in a lipid-free environment were much thicker compared to the protein aggregates grown in the presence of lipids, Figure 3. At the same time, in the presence of negatively charged lipids, hIAPP formed substantially thicker fibrils compared to the aggregates observed in the samples with zwitterionic lipids. We also found that lipids with longer FAs triggered the formation of thicker fibrils compared to the fibrils observed in the presence of lipids with shorter length FAs. These results indicate that net charge of lipids and the length of FAs determine the morphology of hIAPP aggregates. It should be noted that AFM revealed a large amount of small spherical oligomers formed in the presence of DMPC and DPPC as well as in the lipid-free environment that was nearly absent in other samples. These results indicate that zwitterionic lipids facilitate the growth of both oligomers and fibrils, whereas anionic lipids strongly favor hIAPP fibril formation.

**Figure 4:**
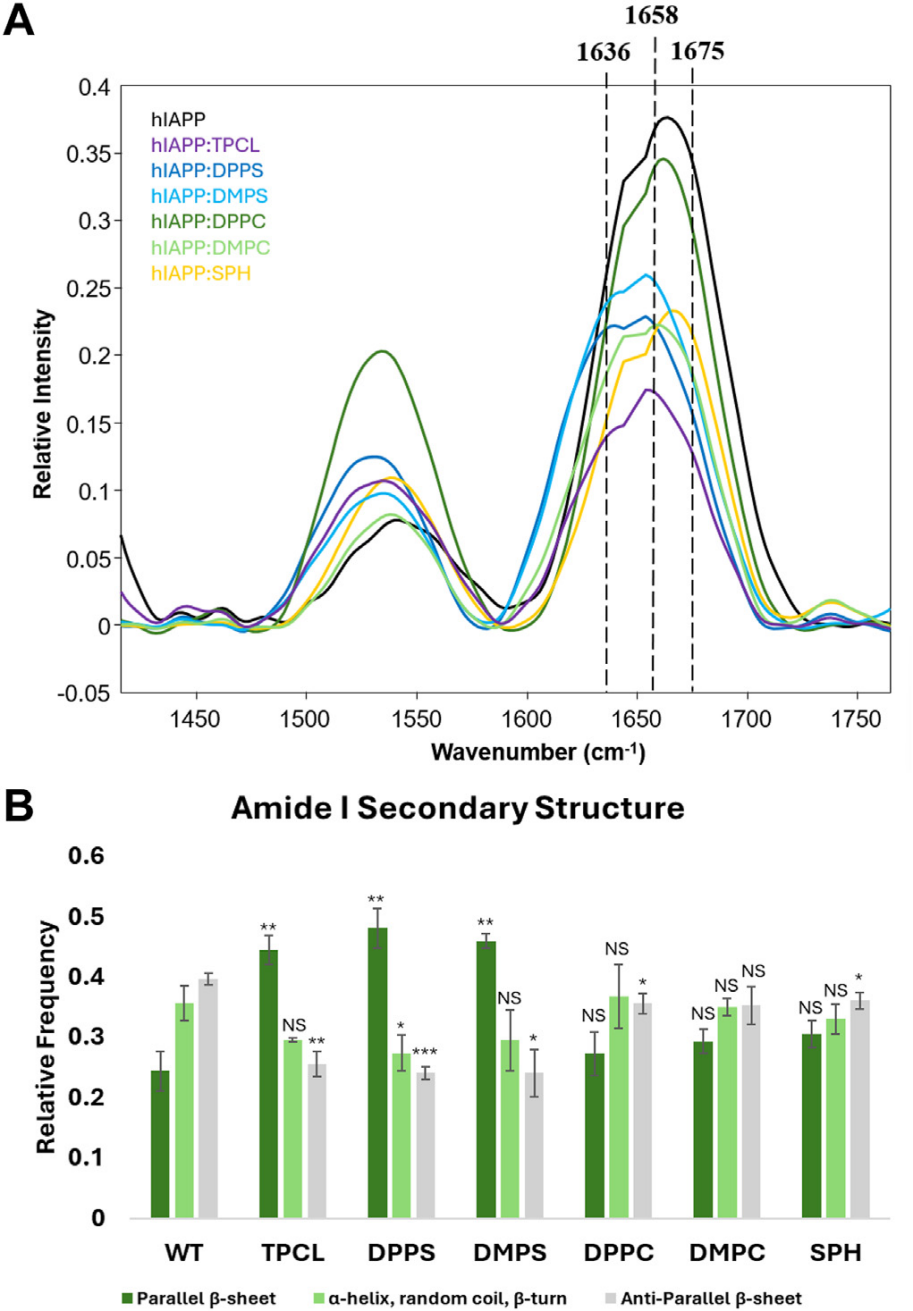
**Lipid membranes change the secondary structure of hIAPP fibrils.***A*, nano-IR spectra acquired from hIAPP fibrils formed in the presence and absence of LUVs composed of different lipids. *B*, distribution of protein secondary structure in the protein aggregates according to the fitting of the amide I band. Spectra are the average of n = 30 spectra. Significance calculated using Student’s *t* test. NS, not significant ∗*p* < 0.05, ∗∗*p* < 0.01, ∗∗∗*p* < 0.001. DMPC, 1,2-dimyristoyl-sn-glycero-3-phosphocholine; DMPS, 1,2-dimyristoyl-sn-glycero-3-phospho-L-serine; DPPC, 1,2-dipalmitoyl-sn-glycero-3-phosphocholine; DPPS, 1,2-dipalmitoyl-sn-glycero-3-phospho-L-serine; LUV, large unilamellar vesicle; SPH, N-octadecanoyl-D-erythro-sphingosylphosphorylcholine; TPCL, 1′,3′-bis[1,2-dipalmitoyl-sn-glycero-3-phospho]-glycerol.

We utilized nano-infrared spectroscopy, also known as AFM infrared (AFM-IR) spectroscopy, to examine the secondary structure of hIAPP fibrils formed in the lipid-free environment and in the presence of lipids. In AFM-IR, metallized scanning probes can be placed on the surface of amyloid aggregates and illuminated by pulse tunable IR light. This causes thermal expansion in the sample of interest that is recorded by the scanning probe. Thermal expansions are converted to IR spectra that can be used to determine the secondary structure of protein aggregates. Specifically, the position of amide I is used to quantify the amount of parallel and antiparallel β-sheet, as well as α-helix, random coil, and β-turn in amyloid fibrils. We found that hIAPP fibrils formed in the lipid-free environment possessed around 25% parallel β-sheet with ∼75% antiparallel β-sheet, α-helix, random coil, and β-turn, Figs. 4 and S1–S3. We also found that hIAPP:TPCL, hIAPP:DMPS, and hIAPP:DPPS fibrils had significantly higher amounts of parallel β-sheet and substantially lower amount of antiparallel β-sheet compared to hIAPP fibrils formed in the lipid-free environment. hIAPP:DPPC and hIAPP:SPH fibrils also possessed a lower amount of antiparallel β-sheet compared to hIAPP fibrils. However, the amount of parallel β-sheet α-helix, random coil and β-turn in these protein aggregates was the same as in hIAPP fibrils. Finally, we found that the presence of DMPC did not alter the secondary structure of hIAPP fibrils. These results indicate that lipids uniquely altered the secondary structure of amyloid fibrils formed by hIAPP, with net charge as the most impactful determining factor.

### Lipids alter cytotoxicity of hIAPP aggregates

**Figure 5:**
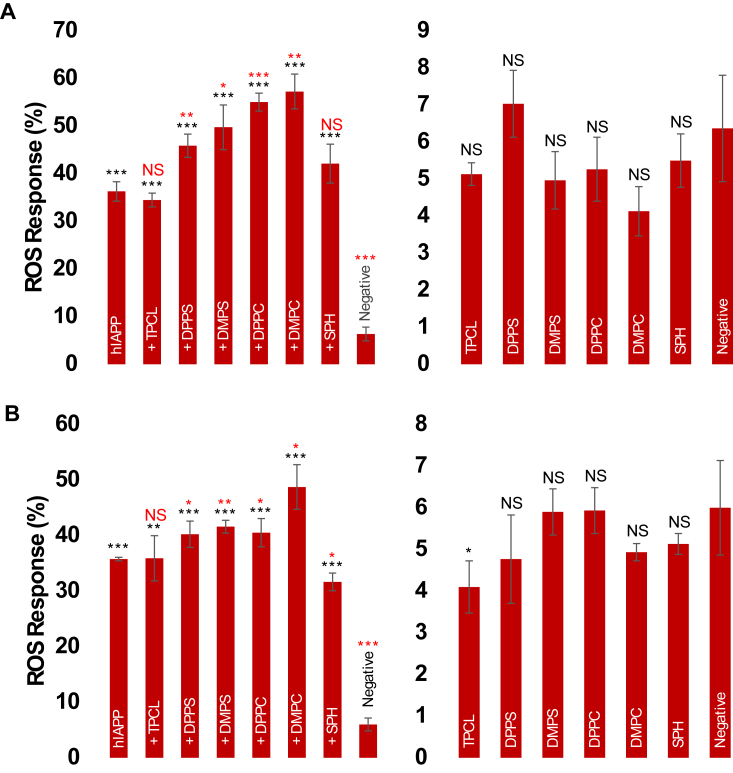
**Lipid membranes alters cytoxicity of hIAPP aggregates.***A* and *B*, ROS assay performed in pancreatic β-cells (*A*) and neurons (*B*) exposed to hIAPP fibrils formed in the absence (hIAPP) and presence of LUVs composed of different lipids (*left*), as well as in the presence of lipids themselves (*right*). Assays were performed in triplicates. Significance calculated using ANOVA with Tukey’s *post hoc* test. ∗*p* < 0.05, ∗∗*p* < 0.01, ∗∗∗*p* < 0.001. DMPC, 1,2-dimyristoyl-sn-glycero-3-phosphocholine; DMPS, 1,2-dimyristoyl-sn-glycero-3-phospho-L-serine; DPPC, 1,2-dipalmitoyl-sn-glycero-3-phosphocholine; DPPS, 1,2-dipalmitoyl-sn-glycero-3-phospho-L-serine; hIAPP, human islet amyloid polypeptide; LUV, large unilamellar vesicle; NS, not significant; ROS, reactive oxygen species; SPH, N-octadecanoyl-D-erythro-sphingosylphosphorylcholine; TPCL, 1′,3′-bis[1,2-dipalmitoyl-sn-glycero-3-phospho]-glycerol.

We utilized rat pancreatic β-cell line BRIN-BD11 to investigate the extent to which lipids altered the toxicity of hIAPP aggregates. Reactive oxygen species (ROS) have been characterized as a marker of amyloid-induced toxicity (28, 29, 30); thus, we utilized a cytoplasmic ROS assay to probe cytotoxicity. We found that hIAPP:DPPS and hIAPP:DMPS fibrils were significantly more toxic, while hIAPP:TPCL aggregates exerted similar cytotoxicity to hIAPP aggregates grown in the lipid-free environment, Figure 5*A*. Moreover, protein aggregates formed in the presence of DPPC and DMPC yielded the highest levels of ROS in pancreatic β-cells (55.01 ± 1.88% and 57.27 ± 3.66% respectively) compared to all other protein aggregates. These results indicate that the chemical structure of lipids determines cytotoxicity of hIAPP aggregates. Our findings also indicate that DMPS and DPPS (49.77 ± 4.7% and 45.9 ± 2.4%) as well as DMPC and DPPC fibrils exerted similar cytotoxicity to pancreatic β-cells, indicating that length of FAs of lipids did not play a significant role in the toxicity of amyloid aggregates.

Type 2 diabetes patients are more susceptible to neurodegenerative diseases, and it has been observed in previous studies that hIAPP can cross the blood–brain barrier and facilitate the aggregation of Aβ (6). In this case, amyloid aggregates formed by hIAPP could propagate in the brain, simultaneously facilitating misfolding and aggregation of amyloid proteins in neuronal tissues. Expanding upon this hypothesis, we investigated cytotoxicity of hIAPP fibrils formed in the presence of lipids and in the lipid-free environment on N27 rat dopaminergic neurons. We found that hIAPP aggregates grown in the presence of DPPS (40.23 ± 2.4%), DMPS (41.6 ± 1.2%), DPPC (40.5 ± 2.55%), and DMPC (48.73 ± 4.0%) exerted significantly higher cytotoxicity compared to hIAPP fibrils formed in the lipid-free environment (35.77 ± 0.3%), Fig. 5, *A* and *B*. At the same time, exposition of neurons to hIAPP:SPH (31.67 ± 1.6%) and hIAPP:TPCL (35.8 ± 4.1%) fibrils resulted in the same and lower levels of ROS compared to hIAPP fibrils. These results further confirmed our findings that the chemical structure of lipids determines cytotoxicity of hIAPP fibrils, and this toxicity appears relatively conserved among tissues. It should be noted that lipids themselves were not toxic to both pancreatic β-cells and neurons.

### hIAPP aggregates grown in the presence of lipids alter the pathway of detoxification

**Figure 6:**
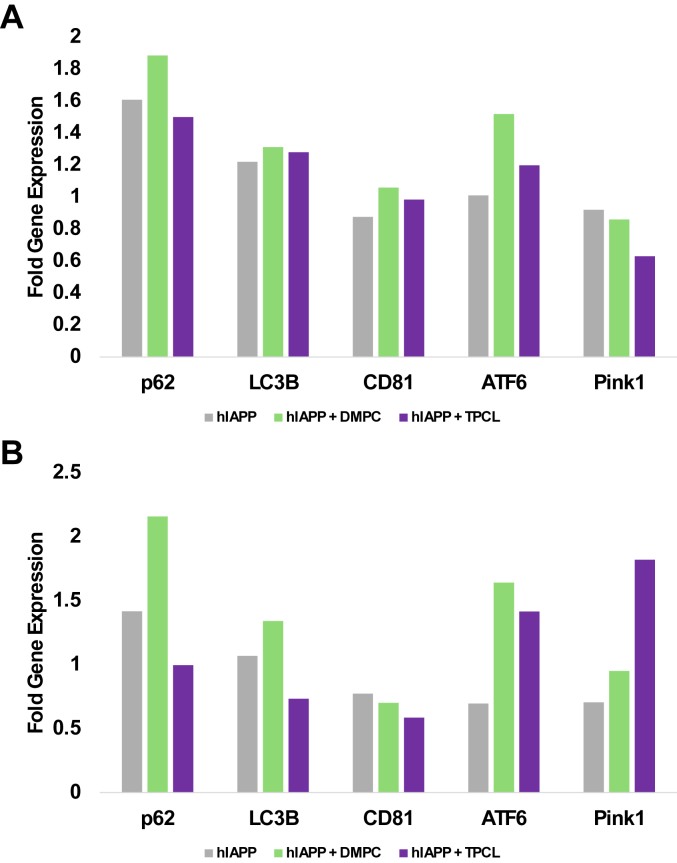
**Lipids alter cellular stress responses to hIAPP aggregates.***A* and *B*, hIAPP aggregate-induced changes in the expression of genes responsible for autophagy (p62 aand LC3B), exocytosis (CD81), UPR response in ER (ATF6), and mitochondria (Pink1) in pancreatic β-cells (*A*) and neurons (*B*). Assays were performed in triplicates. DMPC, 1,2-dimyristoyl-sn-glycero-3-phosphocholine; ER, endoplasmic reticulum; hIAPP, human islet amyloid polypeptide; Pink1, PTEN-induced kinase 1; TPCL, 1′,3′-bis[1,2-dipalmitoyl-sn-glycero-3-phospho]-glycerol; UPR, unfolding protein response.

To reveal molecular mechanisms that determine differences in the cytotoxicity of hIAPP aggregates, we quantified changes in the expression of p62 and LC3, molecular markers of the cell autophagy, Figure 6. Autophagy is an intracellular degradation process that is essential for the survival of eukaryotic cells (31, 32). P62, also called sequestosome 1, is a ubiquitin-binding scaffold protein that colocalizes with ubiquitinated protein aggregates in many neurodegenerative diseases and proteinopathies of the liver (33, 34). LC3 protein is required for autophagosome formation and, therefore, has been widely used to monitor the number of autophagosomes, as well as autophagic activity (35). Moreover, emerging evidence has shown that during selective autophagy, LC3 functions as an adaptor protein to recruit selective cargo to the autophagosome *via* interaction with cargo receptors (36, 37).

Using qPCR, we determined changes in the expression of p62 and LC3 in pancreatic β-cells and neurons exposed to the most (hIAPP:DMPC) and the least toxic (hIAPP:TPCL) protein aggregates, as well as to hIAPP fibrils grown in the lipid-free environment. We found that all amyloid aggregates caused a strong increase in the expression of both p62 and LC3B. These results suggest that pancreatic β-cells and neurons endocytose amyloid aggregates. We also found that hIAPP:DMPC fibrils caused the strongest acceleration in the expression of p62 compared to hIAPP:TPCL and hIAPP. These results indicate that levels of cytotoxicity of protein aggregates are directly linked to the magnitude of their uptake by cells. Our results also show that pancreatic β-cells and neurons did not significantly alter the expression of CD81, a marker of cell exocytosis, as a result of amyloid exposure. These results indicated that both pancreatic β-cells and neurons fail to exocytose protein aggregates. We also found that hIAPP:DMPC, hIAPP:TPCL, and hIAPP fibrils strongly enhanced the expression of ATF6, a marker of unfolding protein response (UPR) in endoplasmic reticulum (ER) of both pancreatic β-cells and neurons. Upon UPR, ATF6 propagates from ER to Golgi where it is cleaved by proteases S1P and S2P that produce an active transcription factor fragment (cATF6). This transcription factor mitigates the ER stress in stressed cells (38, 39). Similar to the expression of p62, we found that hIAPP:DMPC fibrils caused the strongest increase in the expression of ATF6 compared to hIAPP:TPCL and hIAPP. These results indicate that levels of cytotoxicity of protein aggregates are directly linked to the magnitude of UPR in ER.

PINK1 (PTEN-induced kinase 1) is a marker of mitochondrial integrity (40). During UPR response, PINK1 senses mitochondrial dysfunction, which results in the protein accumulation on the outer surface of mitochondrial membrane. PINK1 activates PRKN that plays an important role in eliminating damaged mitochondria *via* mitophagy. qPCR revealed downregulation of the expression of PINK1 in pancreatic β-cells that were exposed to hIAPP:TPCL. At the same time, PINK1 was found to be downregulated in neurons exposed to hIAPP and upregulated in the cells exposed to hIAPP:TPCL. These results indicate that amyloid aggregates could also cause substantial impairment of cell mitochondria in a tissue-specific manner. To further investigate this hypothesis, we utilized JC1 assay.

**Figure 7:**
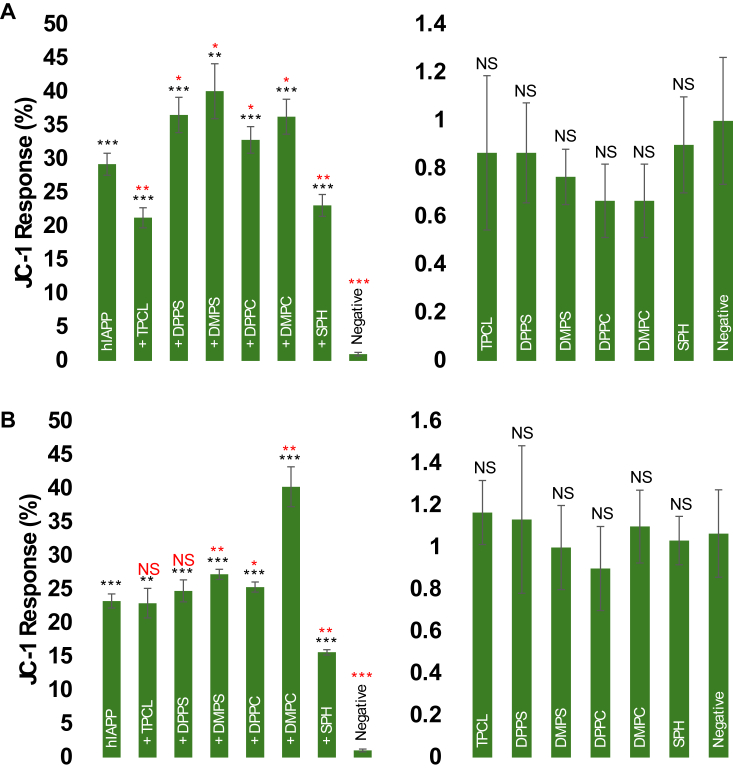
**Lipid membranes alters the extent to which hIAPP aggregates damage cell mitochondria.***A* and *B*, JC1 assay performed in pancreatic β-cells (*A*) and neurons (*B*) exposed to hIAPP fibrils formed in the absence (hIAPP) and presence of LUVs composed of different lipids (*left*), as well as in the presence of lipids themselves (*right*). Assays were performed in triplicates. Significance calculated using ANOVA with Tukey’s *post hoc* test. ∗*p* < 0.05, ∗∗*p* < 0.01, ∗∗∗*p* < 0.001. DMPC, 1,2-dimyristoyl-sn-glycero-3-phosphocholine; DMPS, 1,2-dimyristoyl-sn-glycero-3-phospho-L-serine; DPPC, 1,2-dipalmitoyl-sn-glycero-3-phosphocholine; DPPS, 1,2-dipalmitoyl-sn-glycero-3-phospho-L-serine; NS, not significant; hIAPP, human islet amyloid polypeptide; LUV, large unilamellar vesicle; SPH, N-octadecanoyl-D-erythro-sphingosylphosphorylcholine; TPCL, 1′,3′-bis[1,2-dipalmitoyl-sn-glycero-3-phospho]-glycerol.

JC1 assay revealed that all protein aggregates caused strong impairment of mitochondrial activity in both pancreatic β-cells and neurons. Specifically, hIAPP:DPPS (36.6 ± 2.6%), hIAPP:DMPS (40.13 ± 4.09%), hIAPP:DPPC (32.87 ± 2.0%), and hIAPP:DMPC (36.33 ± 2.6%) fibrils triggered much stronger impairment of mitochondria compared to hIAPP (29.27 ± 1.7%), hIAPP:TPCL (21.3 ± 1.5%), and hIAPP:SPH (23.13 ± 1.6%) aggregates in pancreatic β-cells, Figure 7. Similar results were revealed by JC1 assay on neurons. We found that hIAPP:DMPC fibrils caused the strongest (40.3 ± 3.0%) while hIAPP:SPH fibrils the weakest (15.67 ± 0.4%) impairment of mitochondrial activity compared to all other protein aggregates. It should be noted that lipids themselves were not toxic to mitochondria present in pancreatic β-cells and neurons.

### Dietary supplementation of lipids alters the toxicity of hIAPP *in vivo*

**Figure 8:**
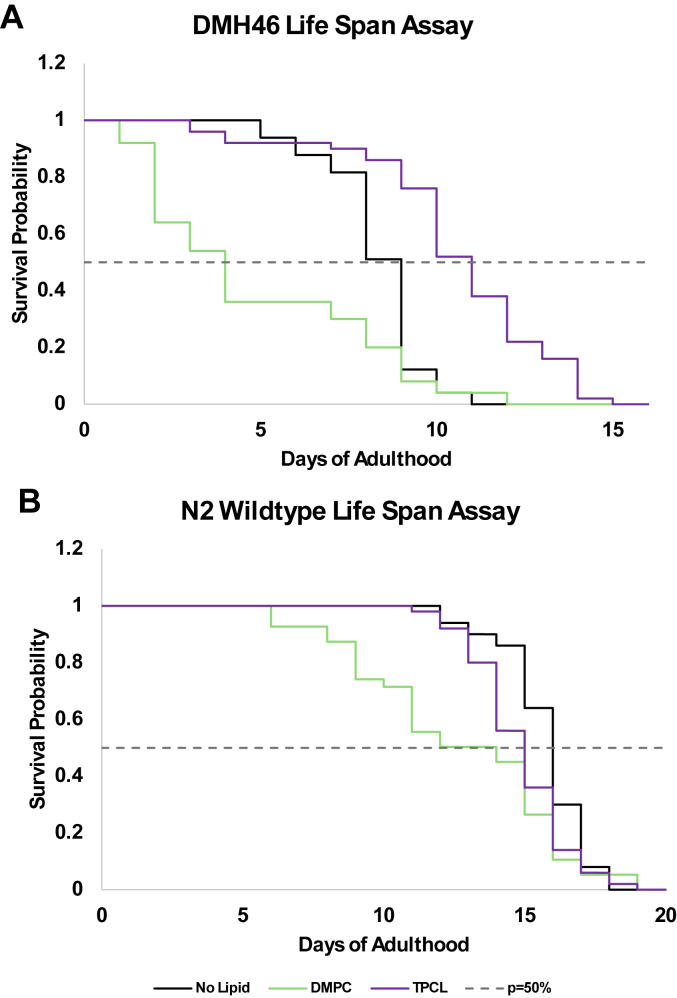
**Lipids alter the toxicity of hIAPP accumulation *in vivo*.***A* and *B*, Kaplan–Meier survival probability curves for DMH46 (*A*) and N2 WT (*B*) *C. elegans* with dietary lipid supplementation. Survival curves based on populations of n = 50 worms. DMPC, 1,2-dimyristoyl-sn-glycero-3-phosphocholine; hIAPP, human islet amyloid polypeptide; TPCL, 1′,3′-bis[1,2-dipalmitoyl-sn-glycero-3-phospho]-glycerol.

The discussed above results suggest that an increase in the concentration of certain phospholipids, such as DMPC, in plasma membranes could drastically increase the cytotoxicity of hIAPP aggregates. To further investigate this hypothesis, we utilized DMH46 strain of *C. elegans* that overexpresses hIAPP (41). In our experiments, *C. elegans* were grown at 25 °C on media enriched with DMPC, a lipid that triggered the formation of the most toxic hIAPP aggregates *in vitro*. We also grew the same strain of *C. elegans* on media with no lipids and on media enriched with TPCL, a phospholipid that did not cause substantial changes in the toxicity of hIAPP aggregates. Lifespan assays were performed to assess relative toxicity of DMPC, TPCL, and no lipid enrichment when hIAPP is overexpressed. We found that DMPC supplementation drastically decreased *C. elegans* lifespan (p50 = 4 days), while TPCL slightly extended (p50 = 11 days) the lifespan of *C. elegans* compared to lipid-free environment (p50 = 9 days), Figure 8*A*. These results demonstrate that an increase in the concentration of dietary DMPC in *C. elegans* corresponded with an increase in the toxicity resulting from hIAPP overexpression. The observed trends in *C. elegans* are strikingly similar to what was observed *in vitro*, further corroborating the hypothesis that specific lipids, such as DMPC, uniquely alter the toxicity of amyloid aggregates.

We utilized wildtype *C. elegans* strain, N2, to investigate possible cytotoxicity of DMPC itself. Although lifespan assays revealed some toxicity for DMPC, comparison of p50 did not reveal any significant effect of DMPC (p50 = 14) and TPCL (p50 = 15) on the lifespan of N2 *C. elegans*, Figure 8*B*. These results indicate that the discussed effects of cytotoxicity of DMPC on *C. elegans* were linked to hIAPP overexpression and protein–lipid interaction.

## Discussion

Our results show how different phospholipid and sphingolipid facilitate hIAPP aggregation. Anionic lipids caused much faster peptide aggregation compared to zwitterionic lipids. These results are in good agreement with experimental results reported by Matveyenka *et al.* and Zhaliazka *et al.* for insulin, lysozyme, and Aβ_1-42_ (42, 43, 44, 45). Specifically, the researchers demonstrated that anionic cardiolipin and phosphatidylserine (PS) at equimolar concentrations drastically accelerated insulin, lysozyme, and Aβ_1-42_ aggregation. It was also shown that zwitterionic lipids fully inhibited aggregation of insulin and lysozyme. This is consistent with previous work done *in silico* by Skeby *et al.* who found that both full-length and truncated hIAPP bound to anionic membranes and accelerated fibril formation (46). However, Zhaliazka *et al.* found that DMPC and other lipids strongly accelerated aggregation of Aβ_1-42_ (44), consistent with what is observed for hIAPP. Previous evidence of lipids below critical micelle concentration catalyzing amyloid formation also explains why all lipids accelerated aggregation and the preferential binding of hIAPP to negatively charged lipids explains the observed increase in acceleration as a function of charge (19, 21). It is possible that the structure and charge of lipids affected the aggregation rate both through membrane surface–based catalysis and through dynamic exchange between free lipids and the lipid membrane. However, the interactions appear to be quite protein specific. Based on these results, we can conclude that the rate of protein aggregation is determined by both the chemical structure of lipids and proteins.

AFM-IR revealed that lipids not only altered the rate of hIAPP aggregation but also modified the secondary structure of amyloid fibrils. These results are in good agreement with experimental results reported by Caillon *et al*. (47). It was found membranes dominated with negatively charged lipids triggered conformational changes in the peptide from disordered to β-sheet-rich, a hallmark of amyloid fibril formation (47). A molecular dynamics study of hIAPP aggregation in the presence of lipid membranes observed a catalyzing effect of membranes promoting the formation of a helical intermediate before commitment to fibril formation, a characteristic of many amyloid proteins (48). Our findings also show that structural differences in amyloid fibrils grown in the presence of different lipids resulted in drastically distinct levels of cytotoxicity that these amyloid aggregates expert to both pancreatic β-cells and neurons. We found that hIAPP aggregates formed in the presence of phosphatidylcholine (PC) and PS with different length of FAs exerted the highest while TPCL and SPH the lowest cytotoxicity. Similar results were recently reported by Dou *et al*. for α-syn (49). It was found that α-syn oligomers and fibrils formed in the presence of both PC and PS were significantly more toxic to neurons compared to α-syn aggregates formed in the lipid-free environment.

Elucidation of molecular mechanisms of lipid-determined cytotoxicity of amyloid aggregates suggested that lipids determine the extent to which hIAPP fibrils are endocytosed and exocytosed by cells, which in turn determines their concentration in cells. We found that exposure of both pancreatic β-cells and neurons to hIAPP aggregates caused an increase in cell autophagy. This indicated an uptake of these aggregates by cells with the aim to degrade them, as corroborated by increased expression of ATF6. At the same time, qPCR revealed decreased expression of CD81, a marker of cell exosomal activity. This indicates that cells are up taking amyloid aggregates but fail to excrete them *via* exocytosis, which results in their accumulation inside pancreatic β-cells and neurons (50). Previously reported results by Matveyenka *et al.* showed that amyloid aggregates damage endosomes (43). This causes their leakage in the cytosol where they increase the production of ROS and damage ER and mitochondria (51). qPCR and JC1 assays confirmed that hIAPP aggregates trigger UPR in ER and impair cell mitochondria. We also found that these effects were determined by lipids that were present during the stage of fibril formation. Specifically, hIAPP aggregates formed in the presence of DMPC caused substantially stronger magnitude of UPR and mitochondrial impairment compared to fibrils formed in the presence of TPCL. The reported results on *C. elegans* confirmed above discussed *in vitro* findings. These results also indicate that cytotoxicity of hIAPP aggregates and consequently, the lifespan of organisms directly depends on their lipid diet. This is consistent with previous studies which indicated that the types of fats consumed by individuals correlates with risk for type 2 diabetes (52, 53, 54, 55). Previous work in transgenic mice further supports this, as Xi *et al.* observed increased hIAPP accumulation associated with high-fat diets (56). Specifically, an increase in the concentration of DMPC in the worm diet caused a drastic decrease in their lifespan. However, this effect was not evident for TPCL, an anionic lipid that did not show high cytotoxicity in the discussed above *in vitro* experiments. Together, this demonstrates that the specific interactions that occur between lipids and hIAPP can be utilized as drug targets to minimize hIAPP aggregation and extend the prognoses of the disease.

*In vivo* studies on *C. elegans* show that an onset of hIAPP toxicity is linked to the presence of DMPC consumed food. *In vitro* studies demonstrate that DMPC and other lipids strongly accelerate hIAPP aggregation which results in the formation of morphologically and structurally different fibrils compared to the lipid-free environment. Furthermore, lipids present at the stage of hIAPP aggregation determine the magnitude of endocytosis of amyloid aggregates by pancreatic β-cells and neurons. Progressive accumulation of hIAPP fibrils in cells caused mitochondria impairment and ER stress ultimately leading to the cell death. These results also suggest that elucidation of lipid–protein interactions could be critically important for the development of novel therapeutic strategies that could prevent the formation of toxic hIAPP fibrils and, consequently, decelerate the onset and propagation of type 2 diabetes.

## Experimental procedures

### Protein sourcing and preparation

Synthetic Human Amylin 1 to 37 was purchased from AnaSpec (Eurogentec) as a lyophilized powder. The protein was treated with 1,1,1,6,6,6-Hexafluoropropa-2-ol at a concentration of 1 mg/ml for 24 h at 4 °C to dissolve any preformed aggregates. The solution was then filtered through a 0.22 um filter, aliquoted, and dried under dry nitrogen gas to form a film of protein. This was freeze-dried for 48 h and then stored at −20 °C for no more than 1 month.

### Liposome preparation

All lipids were purchased from Avanti Polar Lipids. Lipids were dissolved in chloroform and dried under dry nitrogen gas and then freeze-dried overnight. PBS was prewarmed above the melting temperature for each lipid and then added to the dry lipids. Lipid solutions were flash frozen and thawed repeatedly and then sonicated before being extruded through a 100 nm membrane. Lipid vesicle size was confirmed with dynamic light scattering in a DynoPro NanoStar II (Wyatt, Waters Technology).

### Thioflavin T kinetics

Pretreated protein was allowed to reach room temperature and then allowed to come completely into solution in prechilled sterile MilliQ water at a concentration of 100 um. hIAPP and each type of lipid vesicle were added to 1X PBS in a 96-well plate at an equimolar amount equaling a final volume of 95 μl. ThT was added to a final concentration of 50 um. All reagents were kept on ice to prevent premature aggregation. The plate was incubated at 37 °C quiescently for 24 h with fluorescent readings taken at 450 nm excitation and 495 nm emission wavelengths. All conditions were performed in triplicates and fit to Boltzmann’s sigmoidal equation to minimum confidence interval and sum of squared residuals.

### Atomic force microscopy

AFM images were acquired as previously described (57). Samples were dried onto gold-coated silicon wafers, and images were recorded using AIST-NT-HORIBA system in tapping mode and processed using AIST-NT image processing software. Height data were acquired after processing by profile measurement focusing on acquiring height for as many separate fibrils as possible. Fibrils that had varying thicknesses across were measured in multiple locations to accurately represent the whole population, and thicker aggregates that appeared to be multiple fibrils stacked together were avoided in measurements.

### Nano-IR spectroscopy

Samples were prepared by depositing 3 μl of sample on gold-coated silicon substrate and allowed to dry for 15 min before rinsing with DI water and drying by nitrogen gas. AFM-IR imaging and spectral acquisition were acquired by using a nanoIR3 system (Bruker) with a MIRcat-QT laser. AFM imaging was collected through contact mode using AFM tips (PR-EX-nIR2-10 AFM probe, Bruker). The contact-mode tip was optimized using a polymethyl acrylate standard sample for the following wavenumbers: 800-1800 cm-1. Images were taken at a scan rate of 0.3 Hz with a height and width ranging from 1 to 10 μm, resolution of 512 pts for both X and Y parameters, and an I and P gain of 2 and 4, respectively. A total of 30 spectra were collected per sample with a co-average of three for each spectrum acquired, Figs. S1 and S2. The spectra were zapped at the 1648 to 1652 range to remove the artifact caused by the chip-to-chip transition of the instrument. The spectra resolution is 2 cm-1/pt. The spectra were processed using MATLAB as the programming language application.

### IR spectral fitting

Fitting of Nano-IR spectra was performed as previously described (57) in GRAMS/AI Spectroscopy Software. After the amide I region (1570–1800 cm^−1^) was baselined, automated peak identification was performed. Next, fitting was optimized to reach the best possible overlap of the fitted and experimental spectra. Finally, peak areas were determined. Parallel β-sheet was considered from 1616 to 1630 cm^−1^; α-helix and random coil from 1640 to 1670 cm^−1^, and anti-parallel β-sheet from 1690 to 1700 cm^−1^, Fig. S3. All other peaks were discarded from the quantification of the secondary structures. Such peaks could correspond to side chain vibrations (∼1600 cm^−1^) and lipids (1710–1740 cm^−1^).

### Cell toxicity assays

ROS and JC-1 assays were performed as previously described (17). Rat BRIN-BD11 pancreatic cells and rat N27 neurons (Sigma-Aldrich) were cultured in 96-well plates at an average of 30,000 cells per well in RPMI 1640 with 10% FBS. Cells were allowed to adhere and reach around 80% confluence by incubation at 37 °C with 5% CO_2_. Media were replaced with Dulbecco's modified Eagle's medium with 2.5% FBS, and mature aggregates were added to a final concentration of 12 um. Aggregates used for toxicity were prepared the same as in ThT kinetic assays only without the addition of ThT. Based on the ThT kinetic curves, we deemed the aggregates mature after 24 h (approximately 18 h after saturation was reached). Controls were treated with an equal volume of PBS or an equal concentration of lipids. ROS and JC-1 assays were performed 24 h after treatment. All conditions were performed in triplicates.

### qPCR gene expression

qPCR was performed as previously described (58). RNA was extracted from the treated cells using a GeneJET RNA Purification Kit (Thermo Scientific). The concentration of extracted RNA was determined using a NanoDrop One instrument (Thermo Scientific). cDNA synthesis was performed using SuperScript II Reverse Transcriptase (Invitrogen) with random primers (Invitrogen). Specific primers were designed for each target gene with sequences optimized for the specific target genes and to be compatible with qPCR amplification conditions. qPCR reactions were carried out using a C1000 Touch Thermal Cycler (Bio-Rad). Each reaction mixture contained a cDNA template, gene-specific primers, and SYBR Select PCR master mix (Applied Biosystems). PCRs were performed in 35 to 40 cycles, and GADPH was used as a housekeeping gene. Nontemplate controls and positive controls were included in each qPCR run to ensure the accuracy and reliability of the results. Quantification of relative gene expression was calculated using the comparative Ct method (2 −ΔΔCt), where ΔCt represents the difference in threshold cycles between the target gene and the housekeeping gene, and ΔΔCt represents the difference in ΔCt values between the treated samples and the control samples. The relative gene expression levels were calculated and presented as fold changes compared with the control samples.

#### C. elegans

*C. elegans* strain DMH46 was a kind gift from Dr Damien O’Halloran of George Washington University, and N2 wildtype worms were a kind gift from Dr Michael Polymenis of Texas A&M University. Worms were maintained at 20 °C on NGM plates seeded with OP50 *E. coli* and allowed to reach an egg-producing age before age synchronizing as previously described (59). Synchronized worms were allowed to reach day 1 adult age before moving ten worms onto each experimental plate. All experimental plates were made as previously described (59). Lipid supplementation was performed by mixing concentrated stocks with 10× concentrated OP50 before seeding, quickly drying, and UV irradiating. Once worms were transferred to all experimental plates, the incubation temperature was increased to 25 °C to induce expression of hIAPP. Counts of alive and dead worms were taken daily until all worms had died. Survival probability was calculated using the Kaplan–Meier survival curve equation.

## Data availability

Data will be available upon the reasonable request from the authors.

## Supporting information

This article contains supporting information.

## Conflict of interest

The authors declare that they have no conflicts of interest with the contents of this article.
