# Supplementary material for: Lipids determine the toxicity of human islet polypeptide aggregates in vivo
Source: J Biol Chem. 2024 Nov 29;301(1):108029. doi: 10.1016/j.jbc.2024.108029 (PMC11728924; doi:10.1016/j.jbc.2024.108029)
Supplement: Supplementary Information [file mmc1.docx]

**Lipids Determine the Toxicity of Human Islet Polypeptide Aggregates *in Vivo***

Jadon Sitton^1^, Davis Pickett^1^, Axell Rodriguez,^1^ and Dmitry Kurouski^1,2*^

1. Department of Biochemistry and Biophysics, Texas A&M University, College Station, Texas 77843, United States

2. Department of Biomedical Engineering, Texas A&M University, College Station, Texas, 77843, United States

Supplementary Information


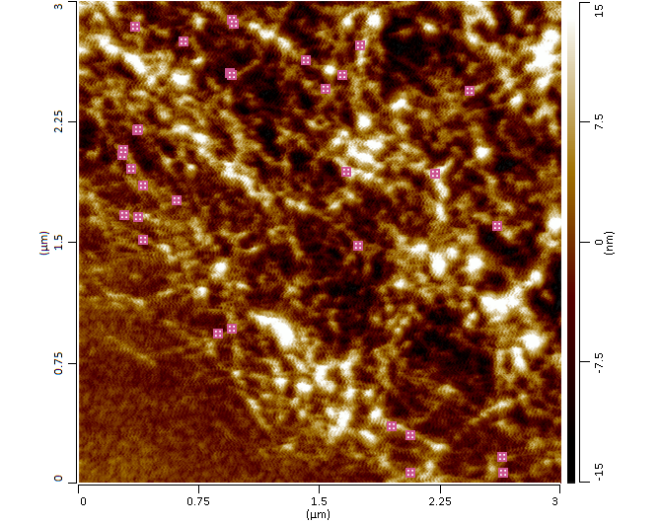

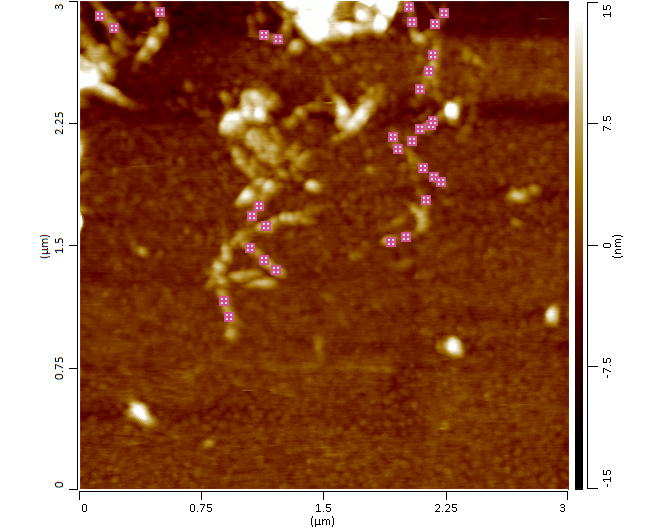


**B**

**A**


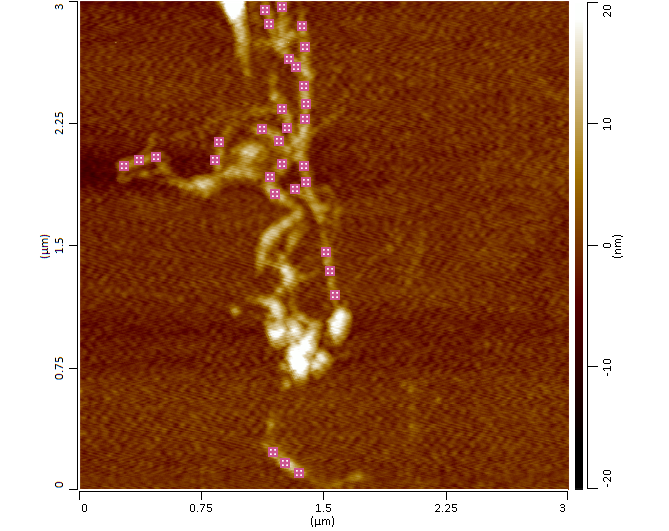

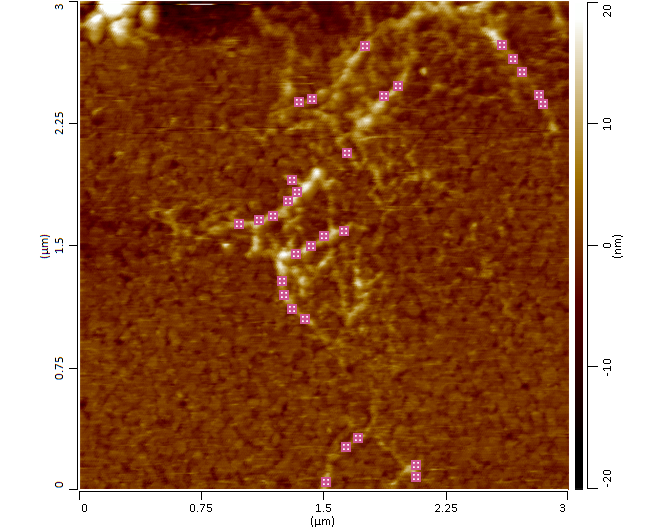


**D**

**C**


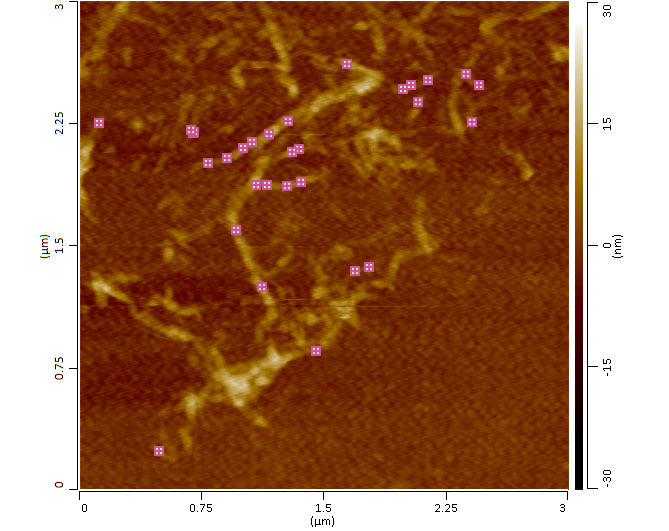

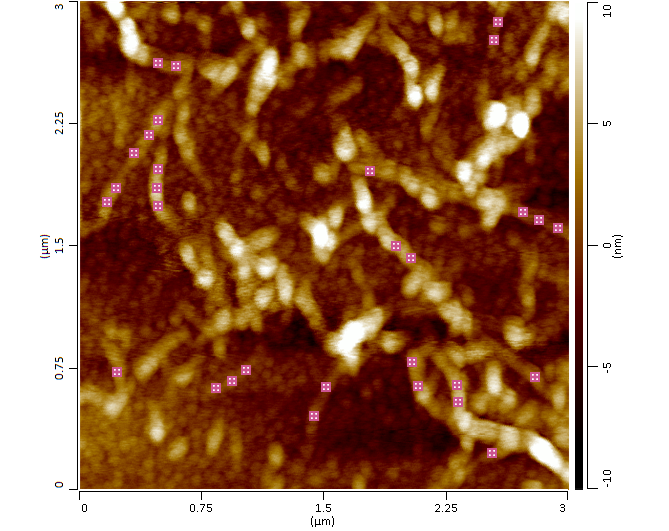


**F**

**E**


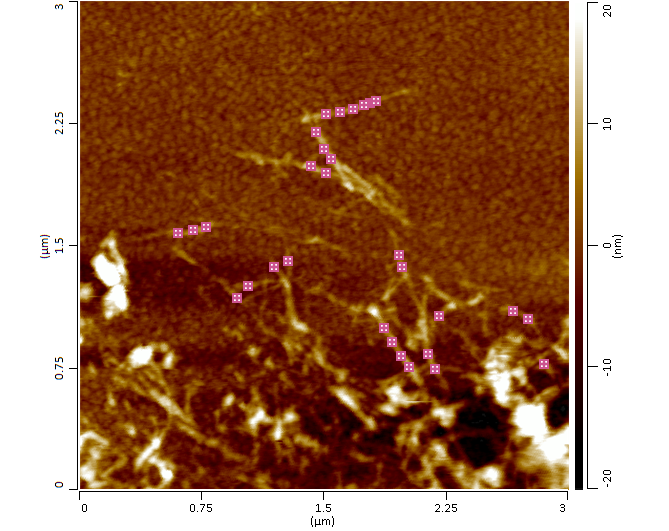


**G**

**Figure S1:** AFM-IR images used for spectra acquisition of hIAPP fibrils formed in a lipid-free environment (A) and in the presence of TPCL (B), DPPS (C), DMPS (D), DPPC (E), DMPC (F), and SPH (G) with point markers.


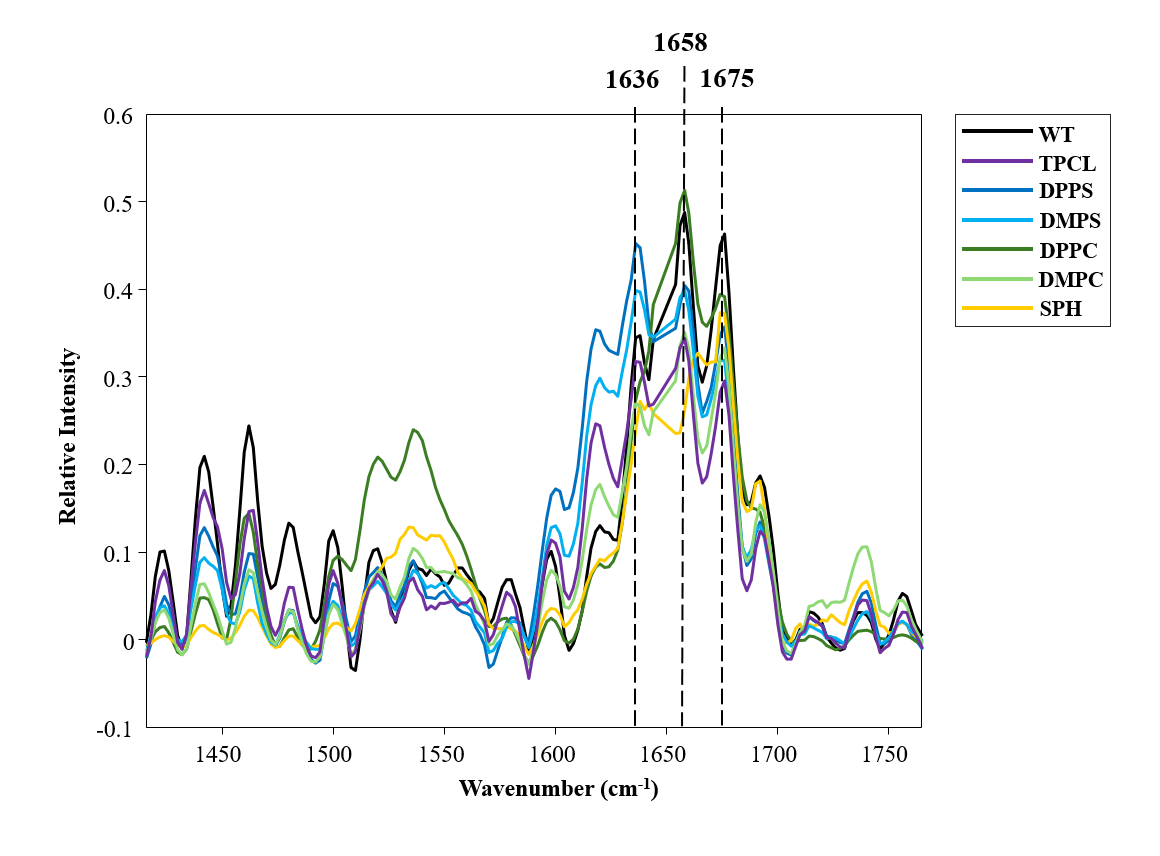


**Figure S2:** Minimally smoothed spectra used for amide I peak fitting.

**A**

**B**

**C**

**D**

**E**

**F**

**G**

**Figure S3:** Peak fitting of coaveraged spectra from hIAPP fibrils formed in a lipid free environment (A) and in the presence of TPCL (B), DPPS (C), DMPS (D), DPPC (E), DMPC (F), and SPH (G).
